# Analysis of Synaptic-Like Microvesicle Exocytosis of B-Cells Using a Live Imaging Technique

**DOI:** 10.1371/journal.pone.0087758

**Published:** 2014-02-04

**Authors:** Aurélie Bergeron, Luca Pucci, Paola Bezzi, Romano Regazzi

**Affiliations:** Department of Fundamental Neurosciences, Faculty of Biology and Medicine, University of Lausanne, Lausanne, Switzerland; The University of Queensland, Australia

## Abstract

Pancreatic β-cells play central roles in blood glucose homeostasis. Beside insulin, these cells release neurotransmitters and other signaling molecules stored in synaptic-like microvesicles (SLMVs). We monitored SLMV exocytosis by transfecting a synaptophysin-pHluorin construct and by visualizing the cells by Total Internal Reflection Fluorescence (TIRF) microscopy. SLMV fusion was elicited by 20 mM glucose and by depolarizing K^+^ concentrations with kinetics comparable to insulin secretion. SLMV exocytosis was prevented by Tetanus and Botulinum-C neurotoxins indicating that the fusion machinery of these organelles includes VAMP-2/-3 and Syntaxin-1, respectively. Sequential visualization of SLMVs by TIRF and epifluorescence microscopy showed that after fusion the vesicle components are rapidly internalized and the organelles re-acidified. Analysis of single fusion episodes revealed the existence of two categories of events. While under basal conditions transient fusion events prevailed, long-lasting episodes were more frequent upon secretagogue exposure. Our observations unveiled similarities between the mechanism of exocytosis of insulin granules and SLMVs. Thus, diabetic conditions characterized by defective insulin secretion are most probably associated also with inappropriate release of molecules stored in SLMVs. The assessment of the contribution of SLMV exocytosis to the manifestation of the disease will be facilitated by the use of the imaging approach described in this study.

## Introduction

Tight control of insulin release from pancreatic ß-cells plays an essential role in the achievement of blood glucose homeostasis. Insufficient insulin supply, resulting from β-cell dysfunction and/or from reduction of the β-cell mass, leads to chronic hyperglycemia and favors the development of different forms of diabetes mellitus, the most common metabolic disorder worldwide [1], [2]. Pancreatic β-cells are located in aggregates of endocrine cells, the islets of Langerhans, which include also α-, γ-, PP- and ε-cells secreting glucagon, somatostatin, pancreatic polypeptide and ghrelin, respectively [3]. Islet cells are all involved in the control of metabolic functions and some of the hormones they release have opposite effects. Consequently, the secretory activity of islet cells has to be highly coordinated. To achieve this goal, islet cells release a wide array of molecules displaying autocrine and paracrine functions. Indeed, besides insulin, pancreatic β-cells secrete Islet Amyloid Polypeptide, Zn^2+^, ATP as well as several neurotransmitters [4], [5] that have putative local signaling functions. Some of these molecules are stored in insulin-containing large dense-core vesicles (LDCVs) and are co-released with insulin [6]. However, others are located in distinct organelles with a diameter of 50–90 nm that closely resemble neuronal secretory vesicles called synaptic-like microvesicles (SLMVs). These secretory organelles contain synaptic vesicle proteins such as synaptophysin and are likely to function as vehicles for storage and release of signaling molecules. [7], [8], [9]. The precise content of β-cell SLMVs is still unclear but they store at least two major inhibitory neurotransmitters, glycine and γ-Aminobutyric acid (GABA) [9]. The physiological role of these neurotransmitters in the islets of Langerhans is only beginning to unfold but GABA has already been shown to inhibit glucagon secretion from pancreatic β-cells and to display autocrine effects on the secretory properties and proliferation of β-cells, suggesting an important contribution in the control of pancreatic endocrine functions [4], [10], [11].

Insulin release can be triggered by different nutrients, hormones and neurotransmitters. Glucose, the main physiological stimulus, enters the cells and its metabolic transformation leads to an increase in the ATP/ADP ratio with the consequent closure of ATP-sensitive K^+^ channels and the depolarization of the plasma membrane. These events cause the opening of voltage-gated Ca^2+^ channels and a rise in the intracellular free Ca^2+^ concentration. The increase of Ca^2+^ combined with the production of other metabolic factors triggers the fusion of LDCVs with the plasma membrane. During the last twenty years, the molecular mechanisms regulating insulin exocytosis have been intensively investigated and many of the components of the machinery governing the secretory process of LDCVs have been identified (for review see [12]). In contrast, the mechanisms and the signaling pathways controlling SLMV exocytosis have been poorly explored because of the characteristics of these organelles. In fact, SLMVs are much smaller than LDCVs rendering more challenging their study by capacitance measurements. Moreover, their content is still poorly defined and several molecules filling SLMVs are also stored in LDCVs. Therefore, it is difficult to follow specifically SLMV exocytosis using standard biochemical techniques or conventional electrophysiological approaches such as amperometry.

In this study, we used a live imaging approach to specifically investigate SLMV exocytosis from pancreatic β-cells. This methodology was used to define the signaling cascades that trigger SLMV release, to identify some of the key components that govern SLMV exocytosis and to determine the kinetics of fusion and recycling of SLMVs.

## Materials and Methods

### Chemicals

Bafilomycin A1, epinephrine, nifedipine and somatostatin were purchased from Sigma (Buchs, Switzerland). Calibration beads were from Molecular Probes (Zug, Switzerland). The antibodies against GFP, Insulin and Synaptophysin were from Chemicon (Zug, Switzerland), Abcam (Cambridge, UK) and Sigma (Buchs, Switzerland), respectively. All other chemicals (unless indicated) were obtained from Invitrogen (Basel, Switzerland).

### Plasmids

The synaptophysin-pHluorin plasmid used for TIRF imaging was generously provided by Dr L. Lagnado (MRC Laboratory of Molecular Biology, Cambridge, U.K.) [13]. The pmCherry-N1 and pEGFP-C2 vector were purchases from Clontech Laboratories (Allschwil, Switzerland). The Tetanus neurotoxin-Tomato construct [14] was kindly provided by Dr. N. Ben Fredj (MRC Centre for Developmental Neurobiology, King’s College London, U.K.). The enhanced GFP-Botulinum neurotoxin C1 construct was obtained from Dr. J. Lang (University of Bordeaux, CBMN, UMR 5248, France). The synaptophysin-mCherry plasmid was produced in our laboratory by subcloning. For this purpose, a plasmid encoding rat synaptophysin 1-EGFP obtained from Dr. Y. Goda (MRC Laboratory of Molecular Cell Biology, Cell Biology Unit, University College London, U.K.) was cleaved between the XhoI and AgeI sites to isolate the coding sequence of rat synaptophysin 1. The DNA fragment was then inserted between the XhoI and AgeI cloning sites of pmCherry-N1.

### Cell Culture and Transient Transfections

The insulin-secreting cell line MIN6 clone B1 [15] was cultured in DMEM-Glutamax medium supplemented with 15% fetal calf serum, 70 µM β-Mercaptoethanol, 50 units/ml penicillin and 50 µg/ml streptomycin. Transient transfection experiments were performed as described [16] using Lipofectamine 2000. Co-transfection experiments were performed using a plasmid DNA ratio of 1∶1.

### Immunocytochemistry

MIN6 cells were plated on glass coverslips coated with 2 mg/ml poly-L-lysine and 33 mg/ml laminin and cultured for 2 days. The cells were rinsed with phosphate-buffered saline (PBS) and fixed in ice-cold methanol for 15 min at room temperature. After two washes in ice-cold PBS, the coverslips were incubated for 10 min with PBS containing 0.5% saponin (PBS-S) and rinsed three times 5 min with PBS. They were then incubated for 30 min in PBS-S containing 1% Bovine Serum Albumin (BSA) and for 1 hour at room temperature in the presence of the primary antibodies diluted in PBS-S plus 1% BSA. The cells were rinsed with PBS, incubated 1 hour with the secondary antibody and mounted for confocal microscopy (Leica SP5 AOBS Confocal Microscope).

### Cell Cultures for Imaging Experiments

MIN6 cells (3.5×10^5^) were plated on glass coverslips coated with 2 mg/ml poly-L-lysine and 33 mg/ml laminin. The cells were transfected with synaptophysin-pHluorin [13], synaptophysin-mCherry or phogrin-mCherry constructs. MIN6 cells were transiently cotransfected with syp-pHluorin and empty pmCherry-N1 or empty eGFP-C2 for controls or cotransfected with syp-pHluorin and plasmids encoding either Tetanus neurotoxin-tomato (TeTx) or enhanced GFP-Botulinum neurotoxin C1 (BoNT/C). Two to four days after transfection, the coverslips were mounted in an experimental chamber at 37°C (Harvard Apparatus) on the stage of a Zeiss Axiovert 200 fluorescence inverted microscope modified to allow epifluorescence (EPI) and evanescence field (TIRF) illumination (Visitron System) [17], [18], [19]. Cells were perfused at 37°C in Krebs-Ringer bicarbonate-HEPES buffer (KRBH: 127 mM NaCl, 4.7 mM KCl, 1.2 mM KH_2_PO_4_,1.2 mM MgSO_4_, 1 mM CaCl_2_, 5 mM NaHCO_3_, 25 mM HEPES, pH 7.4) containing 2 mM glucose (basal solution). They were stimulated with a combination of secretagogues including: KRBH containing 20 mM glucose, 30 mM KCl, 10 µM Forskolin and 100 µM IBMX, KRBH containing 20 mM glucose or 30 mM KCl. Bafilomycin A1 was used at a final concentration of 2.5 µM (diluted with KRBH containing 2 mM glucose) and incubated for 5 min before the stimulus. Nifedipine was used at a final concentration of 10 µM diluted with basal solution, incubated for 15 min before and during all the experiment. In experiments with somatostatin (500 nM) or epinephrine (1 µM), the drugs were diluted in the stimulatory solution and were present throughout the stimulation period.

### Optical Imaging and Data Acquisition

EPI or TIRF illuminations were used for our experiments. In Bafilomycin A1 experiments, EPI and TIRF illuminations were alternated in real time for whole-cell pHluorin fluorescence (pHF) signal measurements in individual cells, in basal condition and upon stimulation. By imaging with excitation light at 488 nm generated by a 488 nm laser (20 mW; Laserphysics) and by a polychromator illumination system (Visichrome), the pHF signal was recorded at 10 Hz through a 100X objective lens (α-plan FLUAR 100X, 1.45 NA, Zeiss, Germany) and filtered with Zeiss filter set 10 (Zeiss) [19]. For TIRF illumination, the expanded beam (488/568 nm argon/krypton multilinelaser, 20 mW) (Laserphysics, USA) was passed through an AOTF laser wavelength selector (VisiTech International) synchronized with a SNAP-HQ CCD camera (Roper Scientific, Germany) under Metafluor software (Universal Imaging, USA) control and was introduced from the high numerical aperture objective lens (α-plan FLUAR 100X, 1.45 NA, Zeiss, Germany). Light entered the coverslips and underwent total internal reflection at the glass-cell interface. In our experimental conditions, penetration depth of TIRF illumination was calculated to be about 90 nm [20]. Light was filtered with a beam splitter (Zeiss filter set 10). Images were acquired at 20–40 Hz. Pixel size 126 nm at binning 2.

### Image Analysis

Video images were digitized with Metafluor and analyzed using the Metamorph software (Universal Imaging, USA) and Origin (OriginLab, USA). The global measurements of the whole-cell pHF intensity during the experiment were obtained by designing a region of interest around the perimeter of the cell surface detectable in the TIRF field. Temporal variations of pHF were expressed as background-subtracted ΔF/F_0._ F_0_ represents the average basal fluorescence level obtained before stimulation and ΔF corresponds to the change in fluorescence occurring during stimulation. The fusion events of SLMVs were manually selected and counted in areas of 6000 pixels (95 µm^2^) on cell surface.

A fluorescent spot was counted as “fusion event” when the pHF signal of a single SLMV increased over basal by ≥4-fold. Quantitative analysis of the subcellular distribution of synaptophysin-pHluorin was carried out using the co-localization module in the Imaris 7.6 software (Bit plane).

### Statistical Analysis

The experiments were analyzed using the SAS statistical package (SAS Inc., Cary NC, USA). Statistical differences were tested by ANOVA. The data on frequency of fusion events at different times were first analyzed by ANOVA, and multiple comparisons of the means were then carried out using the post hoc Dunnett test, with a discriminating p value of 0.05.

## Results

**Figure 1:**
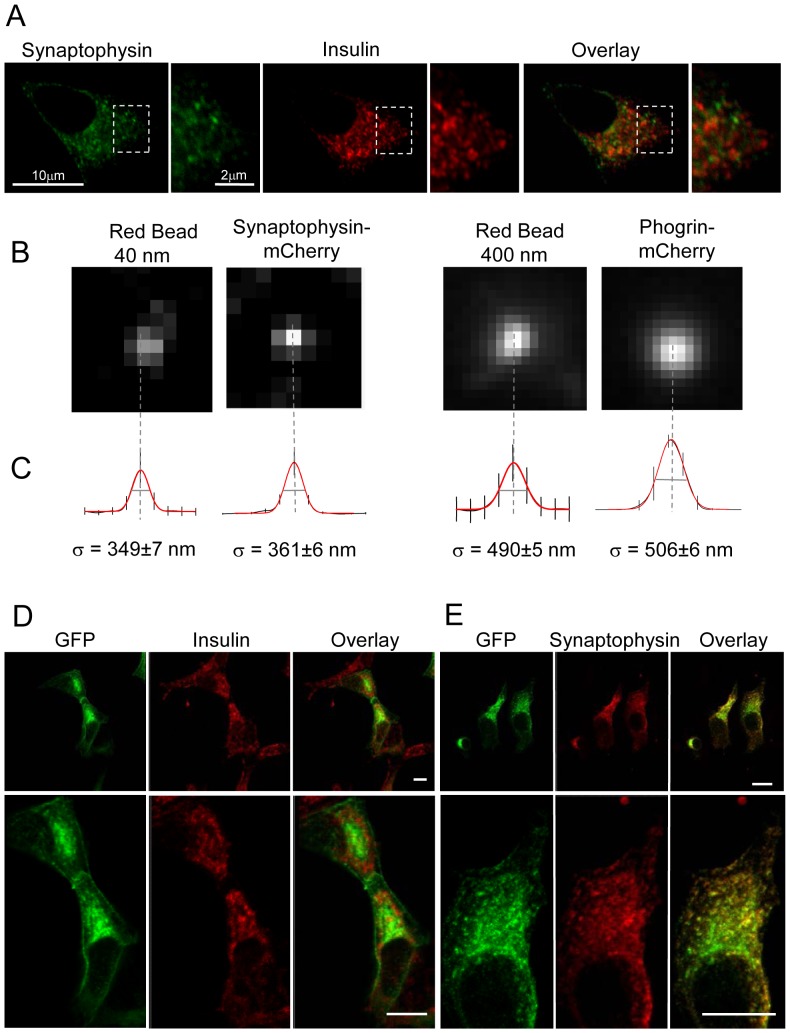
Two types of secretory vesicles in the pancreatic β-cell line MIN6B1. A. Confocal images showing double labeling of endogenous synaptophysin (marker of SLMVs) and insulin (marker of LDCVs) in the pancreatic β-cell line MIN6B1. Lower panels present details of the same images at higher magnification. Note that in the merge images there is no colocalization between the two signals. B. TIRF images of a 40 nm red fluorescent bead, a representative SLMV expressing synaptophysin-mCherry, a 400 nm red fluorescent bead and a representative LDCV. C. Average radial sweeps of 20 TIRF images. Red lines are the means of the measured values. The 40-nm beads are significantly smaller than LDCVs but display apparent sizes similar to SLMVs. In contrast, LDCVs exhibit apparent sizes similar to 400-nm beads. Error bars indicate SD. Bar, 0.5 µm. Intracellular distribution of synaptophysin-pHluorin. D. MIN6 cells were transfected with synaptophysin-pHluorin and immunolabeling performed with antibodies against GFP, endogenous insulin or synaptophysin. Images were acquired with confocal microscope and quantitative analysis carried out using the co-localization module in the Imaris 7.6 software (Bit plane). On the top: the left panel shows the localization of synaptophysin-pHluorin revealed using the GFP antibody (green). The middle panel shows immunolabeling against endogenous insulin (red) and the right panel the merged images. The panels on the bottom present higher magnifications of the top pictures. The analysis of the images shows 8.8±6.8% (n = 5 cells) co-localization between the green and the red signals. E. same experiment as D. but with immunolabeling against synaptophysin (red). The analysis of the images indicates 96.9±2.4% (n = 5 cells) co-localization of the two signals. Bars: 12 µm.

Pancreatic β-cells possess two types of secretory vesicles, insulin-containing large dense-core granules (LDCVs) and synaptic-like microvesicles (SLMVs) [8]. These two categories of secretory vesicles can be readily distinguished by immunocytochemistry and confocal analysis in the pancreatic β-cell line MIN6 using antibodies directed against endogenous insulin and synaptophysin, two specific markers of LDCVs and SLMVs, respectively (Fig. 1A). Individual LDCVs and SLMVs can also be visualized in living cells by transfecting fluorescently labeled constructs that are specifically targeted to each of these secretory organelles. Fig. 1B shows TIRF images where LDCVs and SLMVs are labeled with synaptophysin and phogrin constructs coupled to the fluorescent protein mCherry, respectively. Both LDCVs and SLMVs expressing the chimeric proteins appear as fluorescent spots of different sizes representing secretory vesicles located in the evanescent wave (TIRF illumination) and thus lying close to the plasma membrane. The average diameter of SLMVs obtained by plotting the fluorescence intensity of mCherry against the distance from the center of the spot is close to that of fluorescent beads of 40 nm and LDCVs display sizes similar to beads of 400 nm (Fig. 1C; [19], [21]). These dimensions are in good agreement with estimates obtained by electron microscopy [7].

**Figure 2:**
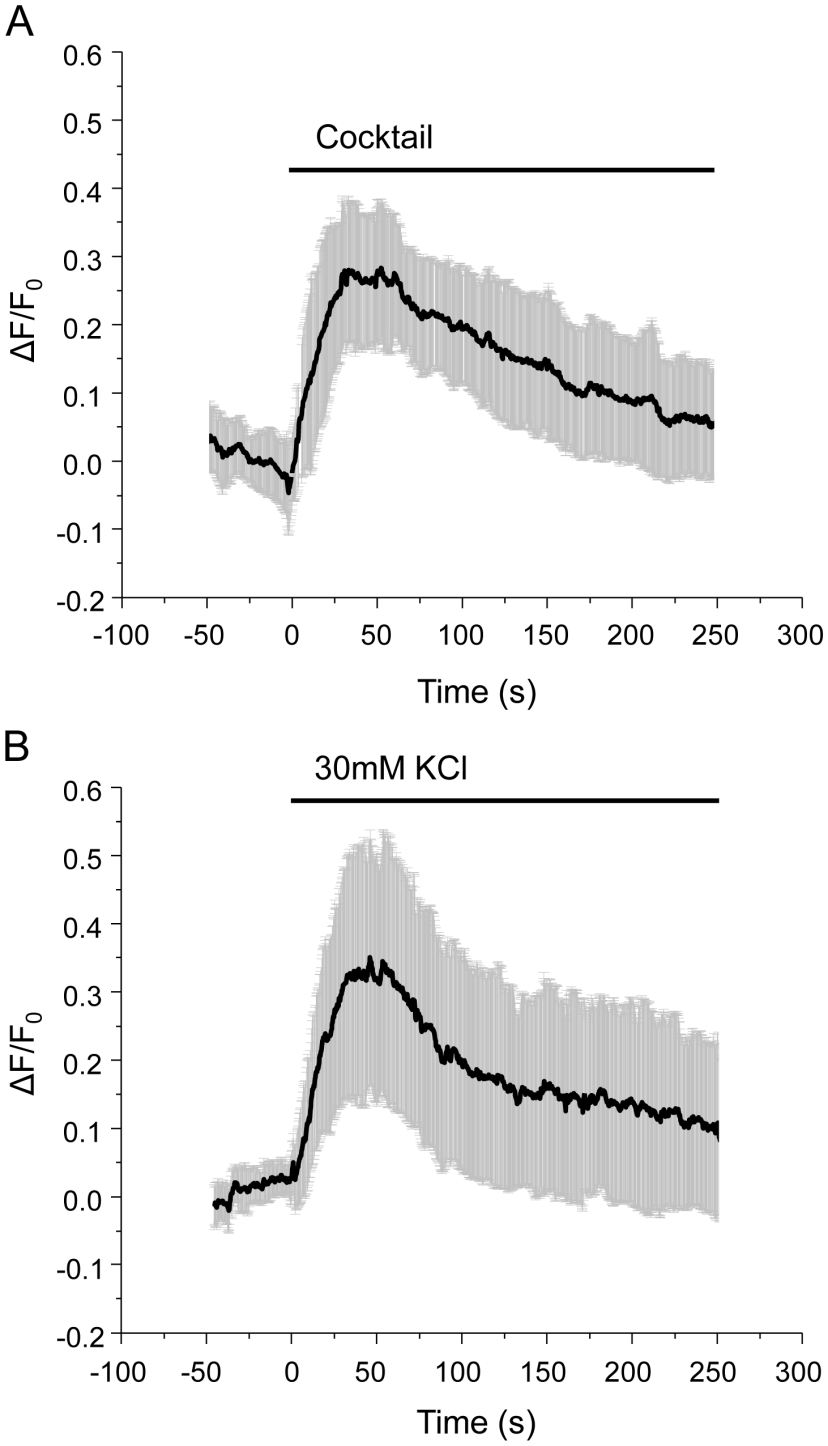
Stimulation of exocytosis and endocytosis of SLMVs in MIN6B1 cells with different secretagogues: whole-cell studies. A. Time-course of cocktail-induced (glucose 20 mM; KCl 30 mM; forskolin 10 µM, IBMX 100 µM) pHluorin fluorescence intensity changes analyzed by TIRF illumination. The curve represents the whole-cell pHluorin fluorescence signal, expressed as ΔF/F_0_ obtained averaging results from 13 cells. B. Same as A. but induced by KCl 30 mM. The curve is obtained by averaging results from 9 cells. Data points are collected every 200 ms and represent mean values ± SD.

**Figure 3:**
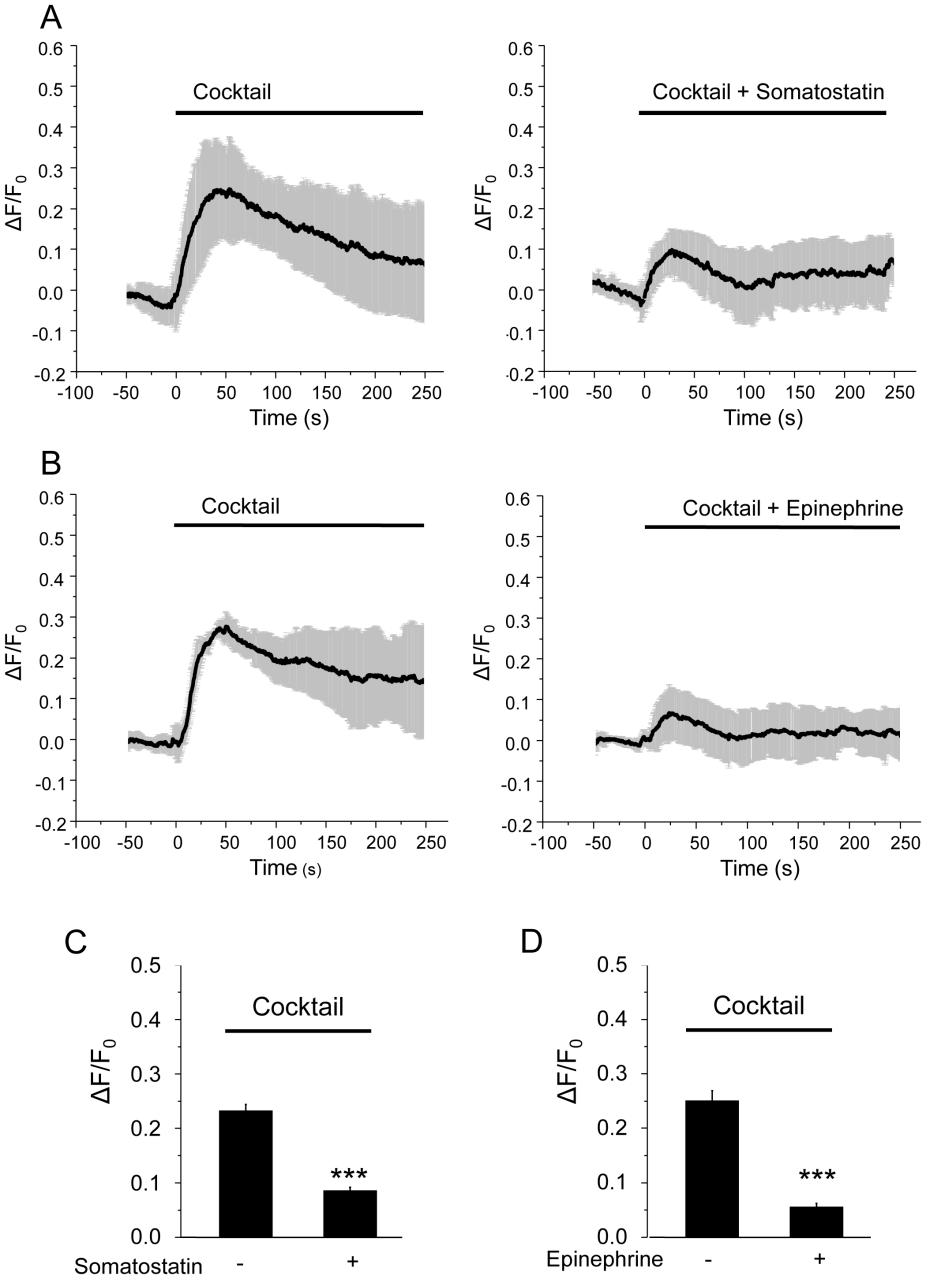
Effect of Somatostatin and Epinephrine on exocytosis of SLMVs. A. Time-course of cocktail-induced (glucose 20 mM; KCl 30 mM; forskolin 10 µM, IBMX 100 µM) pHluorin fluorescence intensity in the absence or in the presence of somatostatin (500 nM) obtained with TIRF illumination. The curve represents the whole-cell pHluorin fluorescence signal, expressed as ΔF/F_0_ obtained averaging results from 5 and 11 cells, respectively. B. same experiment as A. but in the absence or in the presence of epinephrine (1 µM). The curve represents the whole-cell pHluorin fluorescence signal, expressed as ΔF/F_0_ obtained averaging results from 4 and 7 cells, respectively. C–D. Histograms represent the amplitudes (ΔF/F_0_) of the cocktail-induced pHluorin fluorescence intensity change in the absence or in the presence of somatostatin or epinephrine.

While the process underlying the fusion of LDCVs in β-cells has been intensively investigated, the kinetics of SLMV exocytosis and the molecular mechanisms controlling it remain poorly understood. We developed an imaging approach to specifically follow SLMV exocytosis in real time. MIN6 cells were transiently transfected with a chimeric construct consisting of rat synaptophysin fused to a pH sensitive GFP mutant (syp-pHluorin) [13]. In MIN6 cells exogenously expressed syp-pHluorin protein is specifically targeted to SLMVs and not to LDCVs containing insulin (Fig. 1D–E). Indeed, analysis of confocal images using a co-localization software revealed that 96.9±2.4% (n = 5 cells) of the GFP-labelled structures co-localize with endogenous synaptophysin while only 8.8±6.8% (n = 5 cells) of them co-localize with endogenous insulin. Since in the syp-pHluorin construct the fluorescent moiety is located in the luminal domain of synaptophysin and the internal pH of SLMVs is acidic (pH about 5.5), the fluorescence signal emitted before the fusion event is low. However, vesicle fusion with the plasma membrane and exposure of pHluorin to the neutral pH of the extracellular medium (pH about 7.4), results in a sharp rise in fluorescence intensity. This can be readily detected by placing the region of interest (ROI) on the whole-cell perimeter. Exposure of MIN6 cells to a combination of secretagogues including 20 mM glucose, 100 nM IBMX, 1 µM Forskolin and 30 mM KCl led to a transient increase in fluorescence that peaks after about 30–50 sec of incubation (Fig. 2A). Similar observations were made when the cells were incubated in the presence of depolarizing concentrations of KCl alone (Fig. 2B). The recording was stopped after about 300 sec to avoid photo-bleaching. At this time point, the curves did not always return to the basal level, probably because of complete mixing of the SLMVs expressing syp-pHluorin with the plasma membrane and incomplete recapture of the vesicle components [22], [23]. As is the case for the process of insulin secretion, SLMV exocytosis was found to necessitate an increase in intracellular Ca^2+^ concentration. Indeed, the rise in syp-pHluorin fluorescence induced by the secretagogues was prevented by the L-type Ca^2+^-channel blocker nifedipine. Moreover, SLMV fusion was inhibited by hormones and neurotransmitters binding to G_i_-coupled membrane receptors such as epinephrine and somatostatin [24] (Fig. 3).

**Figure 4:**
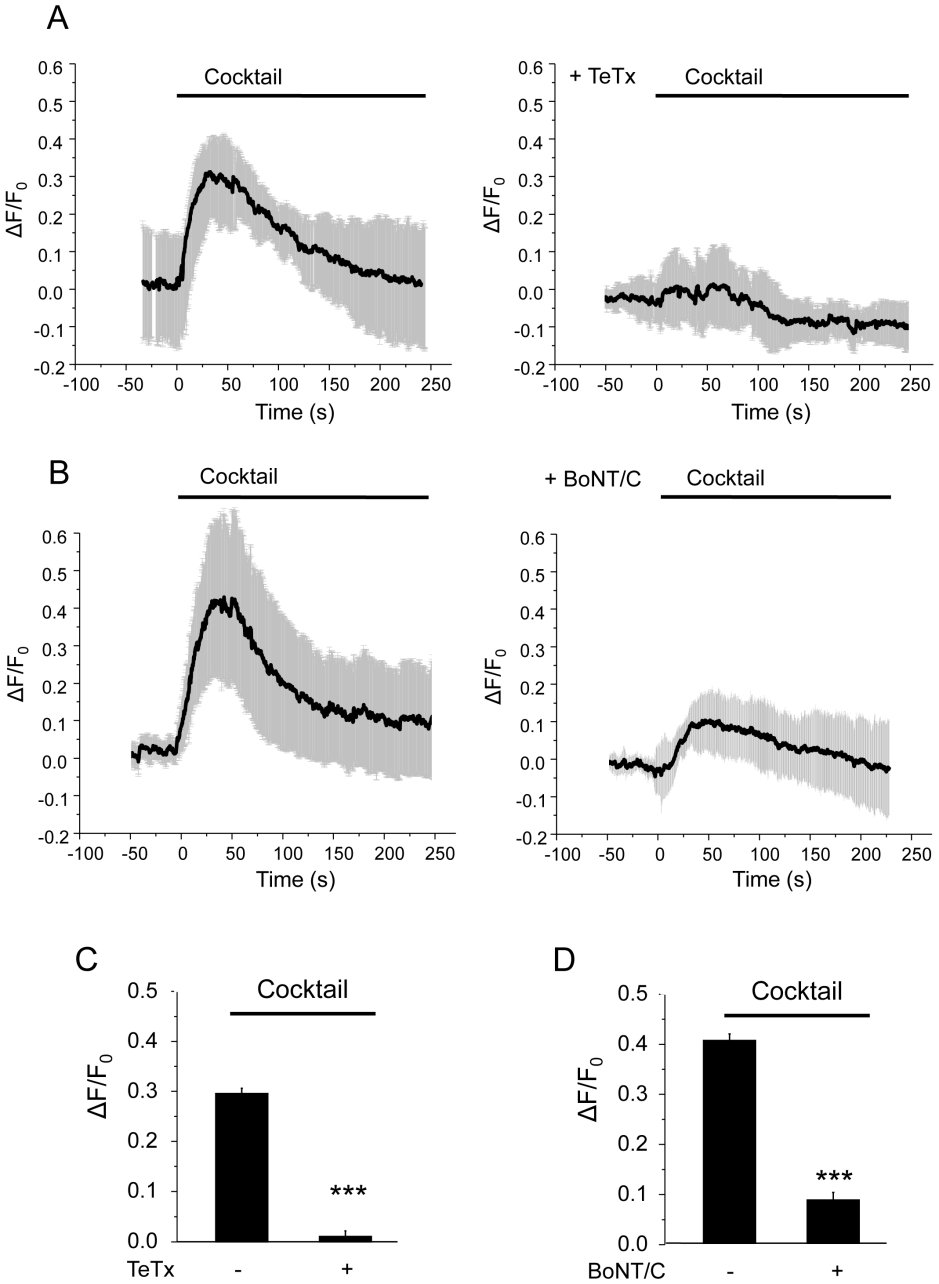
Inhibition of SLMV exocytosis by clostridial neurotoxins. A. Time-course of cocktail-induced (glucose 20 mM; KCl 30 mM; forskolin 10 µM, IBMX 100 µM) pHluorin fluorescence intensity obtained with TIRF illumination in cell transfected with a control plasmid or with tetanus neurotoxin-tomato (TeTX). The curve represents the whole-cell pHluorin fluorescence signal, expressed as ΔF/F_0_ obtained averaging results from 3 and 9 cells, respectively. B. The same as A. but in cells transfected with a control plasmid or with GFP-botulinum neurotoxin C (BoNT/C). Since the GFP-tagged BoNT/C light chain is uniformly distributed in the cytoplasm, the fluorescence signal originating from this construct in the TIRFM evanescent field was negligible. The curve represents the whole-cell pHluorin fluorescence signal, expressed as ΔF/F_0_ obtained averaging results from 5 and 6 cells, respectively. C–D. Histograms represent the amplitudes ΔF/F_0_) of the cocktail-induced pHluorin fluorescence intensity change in cells transfected with control plasmids or with TeTX or BoNT/C.

Insulin exocytosis relies on the formation of complexes including the SNARE proteins VAMP-2 and VAMP-3 (also called cellubrevin) [25]. These proteins have previously been shown to be associated to both LDCVs and SLMVs [25], [26]. To assess whether these SNARE proteins participate in SLMV exocytosis MIN6 cells were transiently co-transfected with the syp-pHluorin construct and with the catalytic subunit of tetanus toxin (TeTx). The light chain of this neurotoxin is a Zn^2+^-dependent protease that specifically cleaves VAMP-2 and -3 [27] and blocks insulin exocytosis [25], [28]. When expressed in MIN6 cells the TeTx light chain was found to be uniformly distributed in the cytosol and to efficiently cleave VAMP-2 (Figure S1 and S2 in File S1). As shown in Fig. 4A, the cells expressing the catalytic subunit of TeTx display an almost complete blockade of the response to the secretagogues, indicating that VAMP-2 and/or VAMP-3 play a central role in SLMV exocytosis. The vesicular SNAREs VAMP-2 and -3 form complexes with their partners located at the plasma membrane. Botulinum neurotoxin serotype C (BoNT-C) has been shown to selectively cleave the plasma membrane SNARE Syntaxin-1 and to inhibit insulin secretion [29], [30]. When expressed in MIN6 cells the catalytic subunit of BoNT-C was homogenously distributed in the cytosol (Figure S1 in File S1) and resulted in Syntaxin 1 cleavage (Figure S2 in File S1). This was accompanied with a substantial reduction in SLMV exocytosis (Fig. 4B), pointing to an important contribution of Syntaxin-1 in the fusion of these vesicles with the plasma membrane.

**Figure 5:**
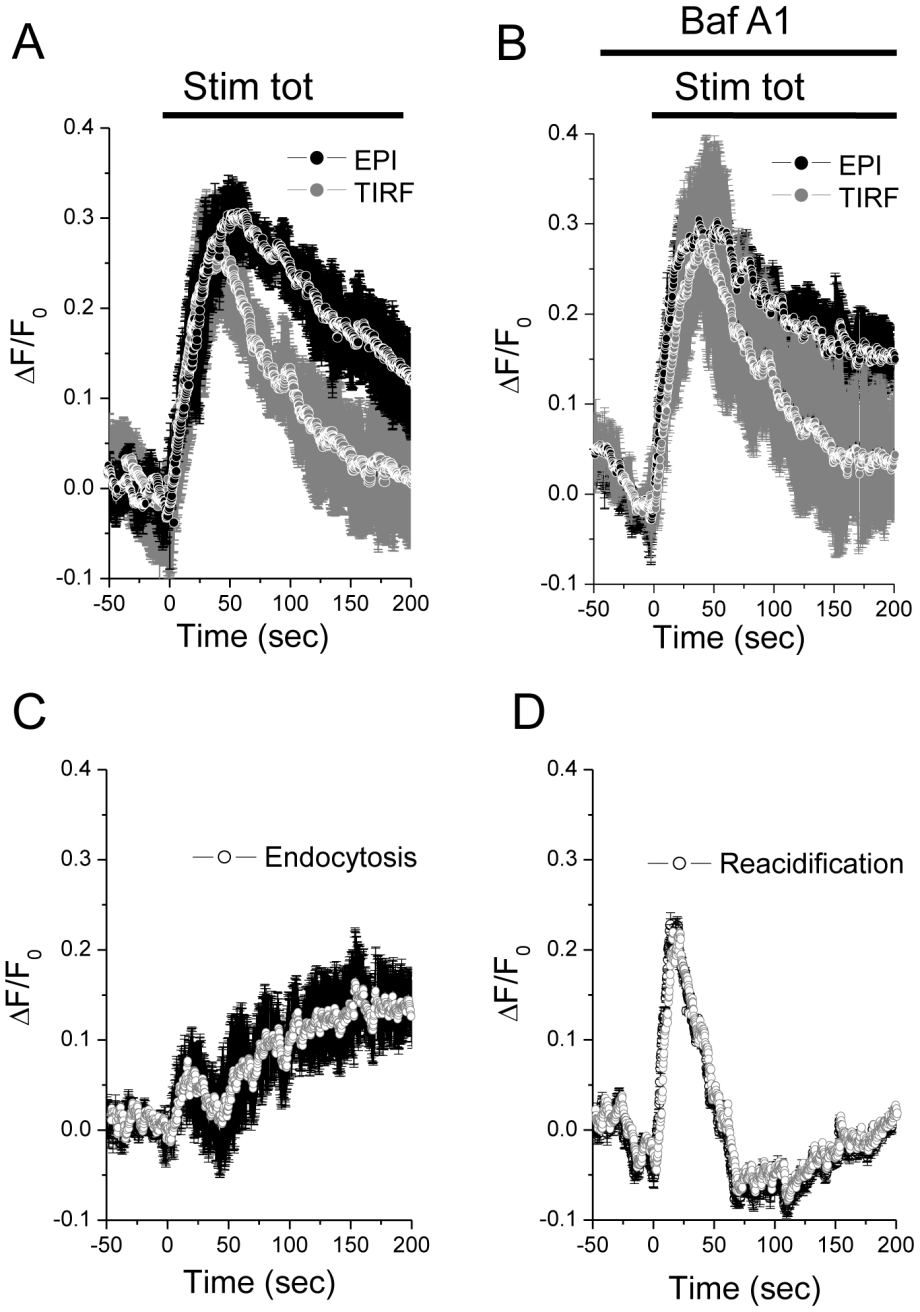
Characteristics of stimulation-evoked exo-endocytosis and reacidification of SLMVs in MIN6B1 cells: whole-cell studies. A. Time-course of cocktail-induced (glucose 20 mM; KCl 30 mM; forskolin 10 µM, IBMX 100 µM) pHluorin fluorescence intensity obtained with TIRF and EPI illumination. The curve represents the whole-cell pHluorin fluorescence signal, expressed as ΔF/F_0_ obtained averaging results from 5 cells. B. Same as A. but in the presence of BafA1 (2.5 µM; n = 4 cells). The rate of spontaneous alkalinization (TIRF 0.08±0.006 ΔF/F_0_; EPI 0.096±0.01 ΔF/F_0_) was calculated in the pre-stimulus period and subtracted from the curves. Not that the curve under EPI represents a cumulative curve of exocytosis. C. Cumulative curve of estimated endocytosis (movement out of the TIRF field) obtained by subtracting the normalized curves of the pHluorin fluorescence signal measured under TIRF and under EPI both in the presence of BafA1 (curves in B). Curves in B were normalized to the maximum fluorescence obtained under EPI and TIRF, respectively in the presence of BafA1 for each cell. D. Cumulative curve of estimated reacidification obtained by subtracting the normalized curves of the pHluorin fluorescence signal obtained under EPI in the absence of BafA1 (curve in A) from the curve in the presence of the drug (curve in B). Curves in A and B were normalized to the maximum fluorescence obtained under EPI in the presence of BafA1 for each cell.

The fluorescence signal detected at the plasma membrane by TIRF microscopy at each time point reflects the combination of different processes including the fusion of SLMVs with the plasma membrane (exocytosis), the internalization of vesicles retrieving the syp-pHluorin construct from the cell surface (endocytosis) and the re-acidification of the vesicle content. The relative contribution of each of these processes can be evaluated by sequentially monitoring the cells by epifluorescence (EPI) and TIRF microscopy. The use of EPI and TIRF microscopy in real time has the advantage of providing a comparative analysis of fluorescence changes in the whole cell and in its sub-membrane compartment, respectively. Moreover, by using two illumination protocols one can obtain information about the kinetics of exocytosis, endocytosis and re-acidification during the application of the secretagogues. Bath application of secretagogues induces in both EPI and TIRF illuminations a rapid and transient increase of syp-pHluorin fluorescence (Fig. 5). In particular, the syp-pHluorin fluorescence under EPI indicates the prevalence of exocytosis (increase of syp-pHluorin fluorescence) over the decay of syp-pHluorin fluorescence after fusion and re-acidification; under TIRF illumination, the rising phase indicates prevalence of exocytosis over the two processes that characterize the endocytic pathway: the fluorescence decay after fusion and re-acidification and the fluorescence decay due to movement of vesicles out of the TIRF illumination [19]. To evaluate the contribution of vesicle re-acidification, we took advantage of the fact that this process relies on the activity of a v-type ATPase [31]. Application of the stimulus in the presence of the v-ATPase inhibitor Bafilomycin A1 [32], produces a change in syp-pHluorin fluorescence that cannot recover because the vesicles retain an alkaline interior (Fig. 5B). Thus, under these conditions the EPI signal reflects only the amount of exocytosis and is independent of the re-acidification. To quantitatively estimate the kinetics of re-acidification we subtracted the curve recorded in the absence of the v-ATPase inhibitor (reflecting a balance between exocytosis and re-acidification) and the EPI curve obtained in the presence of Bafilomycin A1 (representing exocytosis only) (Fig. 5D). The resulting curve permits to directly estimate the kinetic of re-acidification. By analyzing this curve, we found that SLMVs re-acidification occurs in two distinct phases; immediately after the stimulus the process displays an exponential rise for about 20 sec followed by a rapid return to baseline levels. The re-acidification increases again in a more constant and linear manner about 100 sec after the beginning of the stimulus. We then analyzed the TIRF curves. We noticed that Bafilomycin A1 has little or no effect on the rising and decay phases of the syp-pHluorin fluorescence signal. As the contribution of the re-acidification process is eliminated by the presence of the v-ATPase inhibitor, the decrease of the syp-pHluorin signal detected by TIRF illumination reflects exclusively the movement of the SLMVs. We found that the endocytosed vesicles move out of the TIRF illumination before the occurrence of the re-acidification process. The kinetic of this movement can be quantitatively assessed by subtracting the normalized TIRF curve (representing exocytosis and movement) from the normalized EPI curve obtained in the presence of Bafilomycin A1 (representing exocytosis only) (Fig. 5C; see also Text S1 in File S1). Similar to the re-acidification, the endocytosis curve shows a bimodal distribution with a transient peak immediately after the stimulus followed by a steady and slow increase.

**Figure 6:**
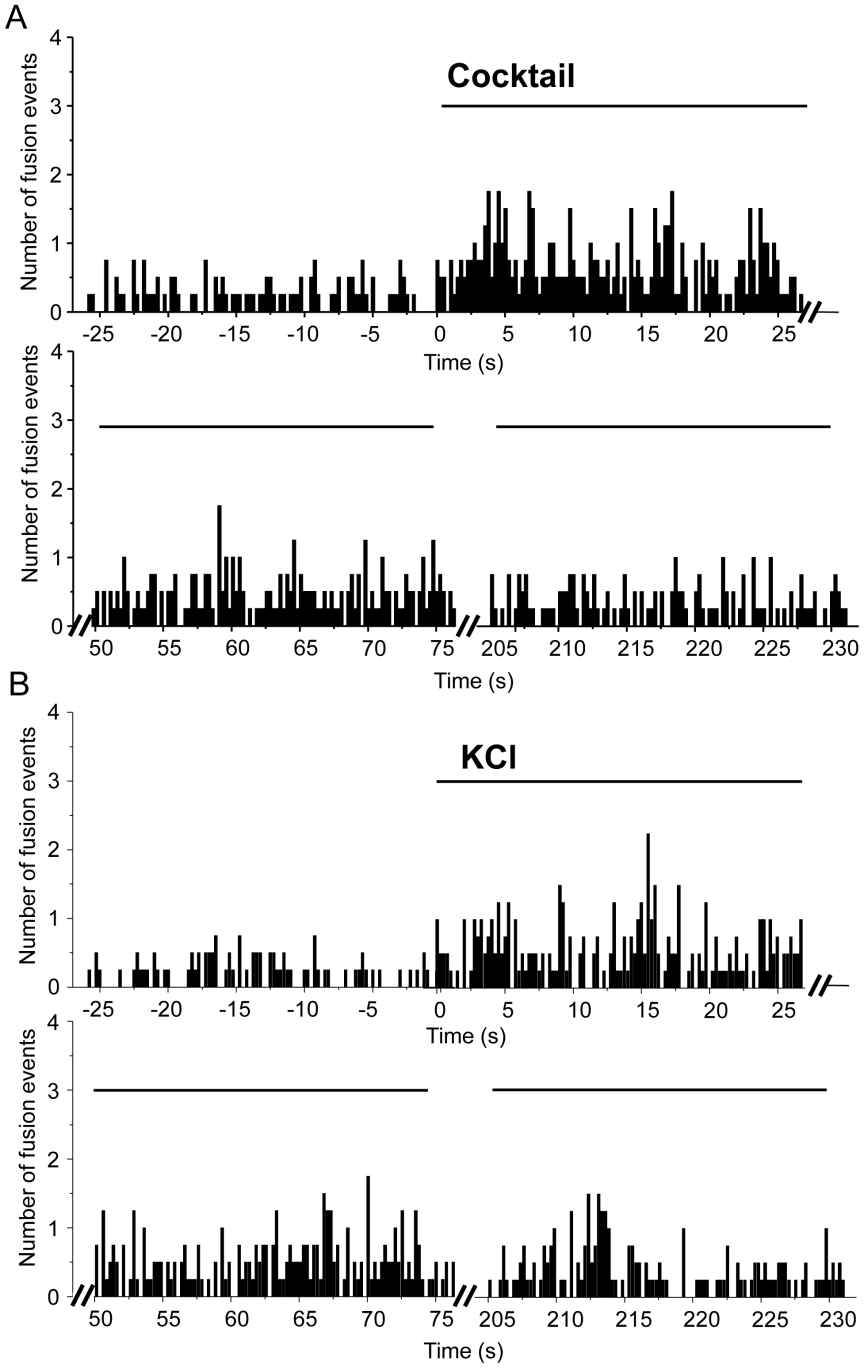
Stimulation triggers SLMV exocytosis in MIN6B1 cells: single vesicle studies. A-B Temporal distribution of the fusion events on a 6000 pixel area (95 µm^2^)/300 ms before and after the bath application of the cocktail (glucose 20 mM; KCl 30 mM; forskolin 10 µM, IBMX 100 µM) or of KCl (30 mM). Note the significant increase in the frequency of pHluorin-mediated fusion events after bath application of both cocktail and KCl (n = 4 cells, from 0 to 75 sec, p<0.05 multiple comparisons, Dunnett test).

**Figure 7:**
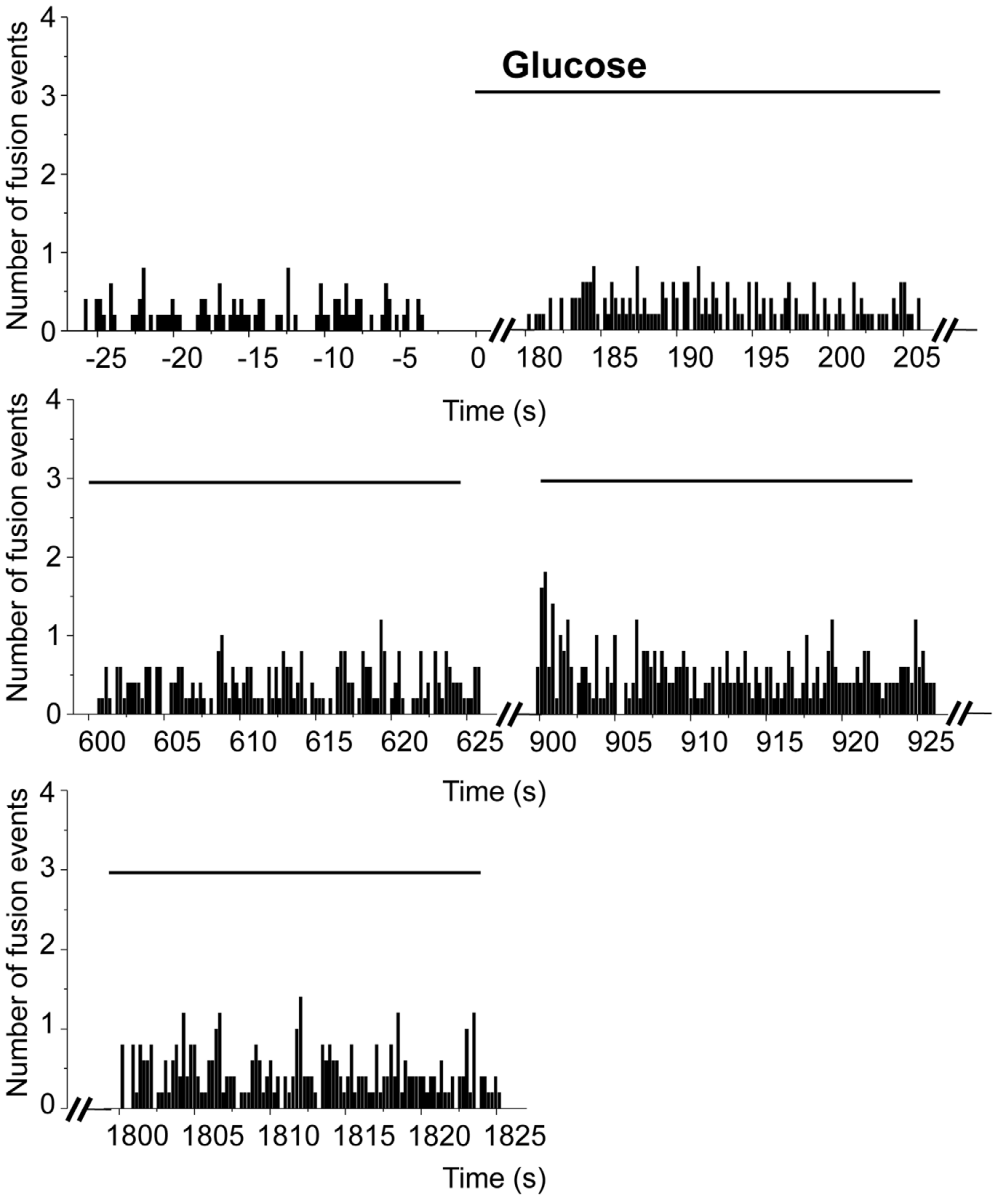
SLMV exocytosis triggered by 20 mM glucose: single vesicle studies. Temporal distribution of the fusion events before and after the bath application of glucose (20 mM). Note the significant increase in the frequency of pHluorin-mediated fusion events after bath application of glucose (n = 5 cells, from 600 sec to 1800 sec, p<0.05 multiple comparisons, Dunnett test).

The analysis of cells expressing syp-pHluorin by TIRF microscopy permits to visualize not only the global fluorescence changes occurring at or very close to the plasma membrane but also to study the exo-endocytosis processes of individual vesicles. Single fusion events are recognized by the appearance of the syp-pHluorin signal in the evanescent field illumination (Fig. 1). By placing the ROI on top of each recognized fusion event we calculated the number of exocytotic events/s (rate of exocytosis) in syp-pHluorin-transfected cells before and after the stimulus. As expected, the number of exocytotic events in cells incubated with a combination of secretagogues or with 30 mM KCl displays a significant increase in the rate of exocytosis during the first 25 sec after stimulation (Fig. 6). It then progressively decreases but remains higher than the number of events preceding the stimulation for at least 5 min. Glucose is the main physiological stimulus of insulin exocytosis [33]. Interestingly, the kinetic of exocytosis was significantly different when cells were incubated with 20 mM glucose (Fig. 7). Similar to insulin secretion the rate of SLMVs exocytosis starts to increase 3 min after the stimulus and remains elevated for at least 30 min. This is consistent with the signaling pathway underlying stimulus-secretion coupling elicited by glucose. Indeed, glucose is first metabolized by the cells to generate ATP. This, in turn, causes closure of ATP-sensitive K^+^ channels, membrane depolarization and Ca^2+^ entry through voltage-gated Ca^2+^ channels [33].

**Figure 8:**
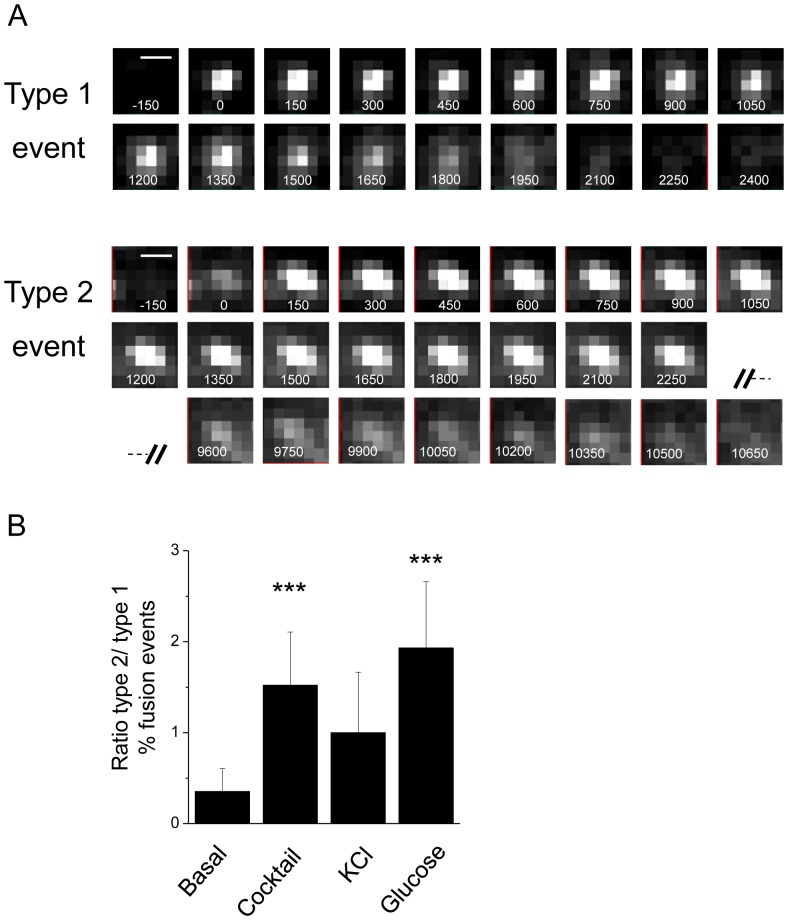
Two modes of exo-endocytosis: single vesicle studies. A. Serial TIRF images illustrate typical type 1 and type 2 fusion events of SLMVs expressing syn-pHluorin. Time 0 represents the fusion event. Each frame corresponds to 150 milliseconds. The syn-pHluorin labelled vesicle start to fuse with the plasma membrane at 0. B. Histograms representing the ratio between type 1 and type 2 fusion events under basal conditions or in the presence of different stimuli (cocktail, KCl and glucose) (n = 5 cells, t-test, p<0.05).

Beside the quantitative assessment of the number of fusion events, monitoring of the cells by TIRF microscopy permits a detailed analysis of the dynamics of the fusion process. This led us to distinguish two categories of fusion events: transient events characterized by a rise in fluorescence that disappears within about 2 sec (Type 1) and long lasting events in which the fluorescence signal persists at the membrane for several seconds and fades only after about 10 sec (Type 2) (Fig. 8A). Under resting conditions close to 70% of the fusion events were of Type 1 while 30% were classified of Type 2 (Fig. 8B). This ratio was significantly modified after stimulation of the cells. Indeed, 50% or more of the fusion events observed upon incubation of the cells in the presence of secretagogues were of Type 2 (Fig. 8B).

## Discussion

Pancreatic β-cells play a pivotal role in the control of body metabolism. A detailed knowledge of the physiology of these cells is essential to understand the causes of defective insulin release in pre-diabetic and diabetic conditions and to favor the development of new therapeutic approaches for the treatment of this disease. Insulin is stored in LDCVs and is released in response to a variety of nutrients, hormones and neurotransmitters [33]. However, β-cells release many other signaling molecules that are believed to modulate their functional status and to affect their secretory activity as well as their survival and proliferation rate. Among them, GABA has attracted considerable attention after the demonstration that in rodents this neurotransmitter inhibits glucagon secretion from pancreatic β-cells and displays autocrine effects on the secretory properties and proliferation of β-cells [4], [10], [11]. Recent studies confirmed a role for GABA in the control of pancreatic endocrine functions in human islets [34] and revealed that the GABA signaling system is compromised in islets obtained from Type 2 diabetic individuals [35]. Many non-peptide signaling molecules released by β-cells, including a fraction of GABA, are stored in SLMVs [8]. In contrast to LDCV exocytosis, which has been intensively investigated, the mechanisms governing exo-endocytosis processes of SLMVs are still largely unknown. This lack of information is mainly due to difficulties in studying these organelles with standard techniques. Indeed, their small size renders conventional capacitance measurements technically demanding and the repertoire of molecules residing inside the SLMVs is still not fully established. Recent studies performed with cell-attached membrane capacitance suggested that SLMVs undergo regulated exocytosis as neuronal synaptic vesicles. Similar to insulin secretion, SLMVs were reported to fuse with the plasma membrane in response to glucose and to an increase in cytosolic calcium [36]. Although these studies provided an initial characterization of the properties of SLMVs in β-cells, a number of key questions regarding the kinetics of the exocytotic and recycling processes and the molecular determinants governing them remained unsettled.

In this study, we developed a strategy to specifically monitor SLMV exocytosis from insulin-secreting cells. In recent years, membrane tracer approaches combined with sensitive fluorescent imaging techniques have emerged as superior tools to study the fate of specific organelles and to monitor secretion processes in real time. In particular, the use of chimeric constructs including a pH sensitive form of GFP (termed super-ecliptic pHluorin) is recognized as the state-of-the-art approach for imaging secretion processes in living cells [37]. Indeed, the attachment of pHluorin to the interior of various vesicle proteins such as synaptobrevin [32], synaptophysin [13] or VGLUT1 [19], [38] allowed the analysis in real-time of exocytosis and recycling of specific populations of secretory organelles. Here we took advantage of this life imaging approach to assess the effect of several secretagogues and to dissect the mechanisms underlying SLMV fusion in pancreatic β-cells. Our data indicate that in β-cells SLMV exocytosis occurs in a regulated fashion and is triggered by the same stimuli that elicit insulin exocytosis. As is the case for LDCVs, fusion of SLMVs with the plasma membrane involves opening of voltage-gated L-type Ca^2+^ channels and a consequent increase in intracellular Ca^2+^ concentration. We also provide data indicating that SLMV exocytosis is negatively controlled by agonists that bind to Gi-coupled receptors and potently inhibit insulin secretion such as epinephrine and somatostatin [24]. These data confirm and further extend the results obtained using capacitance measurements in INS-1 and primary rat β-cells [36].

In our study, we also investigated the molecular mechanisms underlying the fusion of SLMVs with the plasma membrane. Using clostridial toxins we were able to show that SLMV exocytosis in β-cells relies on the activity of VAMP-2/−3 and Syntaxin-1, a group of SNARE proteins playing a central role in neuronal transmitter release and in insulin secretion [39], [40]. The level of these proteins has been shown to be reduced under physiopathological conditions and in diabetes animal models [41], [42]. Moreover, VAMP-2 and Syntaxin-1 are strongly diminished in pancreatic islets isolated from Type 2 diabetic patients [43]. Thus, under physiopathological conditions in which glucose metabolism and Ca^2+^ signaling are defective and/or the expression of VAMP-2 and Syntaxin-1 is reduced the release of compounds located in SLMVs is likely to be altered. This may potentially contribute to defective β-cell function in pre-diabetic and diabetic conditions.

The imaging technique used in this study permitted to characterize the dynamics of the exocytotic and recycling processes of β-cell SLMVs with unprecedented details. Although our approach focuses on a specific cell area that is in contact with the coverglass, our observations are likely to be representative of the behaviour of SLMVs in the rest of the cell. Indeed, although with the limitation of the imaging techniques studying secretion in the cellular area in contact with the coverglass, we were able to show that endocytosis and re-acidification of the vesicles are initiated immediately after the fusion of SLMVs with the plasma membrane and display bimodal kinetics. As is the case in neuronal cells, SLMVs are recycled rapidly and are likely to be again available for subsequent fusion events in a relatively short time. Compared to endogenous vesicular proteins that remain clustered at the site of fusion, pHluorin constructs tend to diffuse more in the plasma membrane [22], [23]. Thus, it is possible that the recapture of the SLMV components is even more efficient than detected by our experimental approach. In view of the fact that β-cells contain around 3′500 SLMVs and only a very small fraction of them is undergoing exocytosis even under maximal stimulation, a shortage of these secretory organelles is very unlikely under physiological conditions. Interestingly, both endocytosis and recycling of SLMVs display a bimodal pattern. The two distinct phases of endocytosis and recycling observed may possibly reflect the existence of different vesicle pools: one readily available for fusion and rapidly recycled and another mobilized only upon prolonged exposure to secretagogues. This issue will need to be investigated in more details in future studies.

Careful analysis of individual fusion episodes revealed the existence of at least two types of exocytotic events: short short-lived events that predominate under basal conditions and long-lasting events more frequent under stimulatory conditions. At present, the functional significance of these two modes of exocytosis remains to be elucidated and several explanations can be envisaged. One possibility is that short living fusions correspond to events in which the vesicle and the plasma membrane components are not fully merging. Under these conditions, closure of the fusion pore could permit immediate re-acidification of the vesicle content and rapid recycling. In contrast, long-lasting fusions may represent exocytotic events in which the vesicle and the plasma membrane are fully mixing. In this case, recapture of the vesicle components and re-acidification may require a longer period. Another possible explanation for our findings is that when secretion is slow (basal conditions) most of the vesicles can be rapidly endocytosed and recycled while under strong stimulatory conditions the capacity of the cells to retrieve the vesicle components from the plasma membrane becomes rate-limiting. Under these circumstances the vesicle components would accumulate at the cell surface leading to a persistently elevated syp-pHluorin fluorescence signal.

In conclusion, the imaging approach described in this study allowed careful analysis of the molecular determinants and of the kinetics of exocytosis and recycling of β-cell SLMVs. We obtained new information about the signaling cascades and the molecular components involved in SLMV exocytosis and were able to monitor the characteristics of single fusion events. In the future, the use of our methodology will help clarifying the precise physiological role of these organelles and will permit to determine whether inappropriate release of molecules stored in SLMVs contributes to β-cell dysfunction occurring in pre-diabetic and diabetic state.

## Supporting Information

File S1 Figure S1) The transfected catalytic subunits of clostridial toxins are uniformly distributed in the cytosol of MIN6 cells. Figure S2) Expression of the light chains of TeTx and BoNT-C in MIN6 cells results in efficient cleavage of VAMP2 and Syntaxin 1. Text S1) Detailed explanation of the procedure used for the analysis of the different parameters of SLMVs recycling.
(PDF)
