# Supplementary material for: Analysis of Synaptic-Like Microvesicle Exocytosis of B-Cells Using a Live Imaging Technique
Source: PLoS One. 2014 Feb 4;9(2):e87758. doi: 10.1371/journal.pone.0087758 (PMC3913683; doi:10.1371/journal.pone.0087758)
Supplement: File S1 — Figure S1) The transfected catalytic subunits of clostridial toxins are uniformly distributed in the cytosol of MIN6 cells. Figure S2) Expression of the light chains of TeTx and BoNT-C in MIN6 cells results in efficient cleavage of VAMP2 and Syntaxin 1. Text S1) Detailed explanation of the procedure used for the analysis of the different parameters of SLMVs recycling. (PDF) [file pone.0087758.s001.pdf]

Supporting information

Figure S1

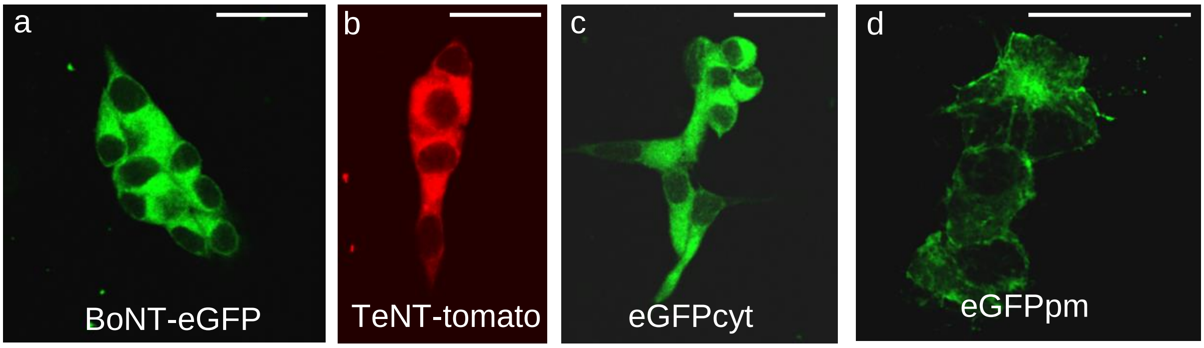

Figure S1) The transfected catalytic subunits of clostridial toxins are uniformly distributed in the cytosol of MIN6 cells. a-d Confocal images showing MIN6 transfected with BoNT-eGFP (a), TeNT-tomato (b), eGFP alone (remaining in the cytosol) (c) and eGFP coupled to the PH domain of phospholipase C that binds to the plasma membrane (Waselle et al. Mol Endocrinol 19: 3097-106 (2005)) (d). Bars: 20 μm. These observations exclude that the effects of the toxins on SLMVs exocytosis are caused by non-specific accumulation at the plasma membrane and by alterations of the membrane properties.

Figure S2

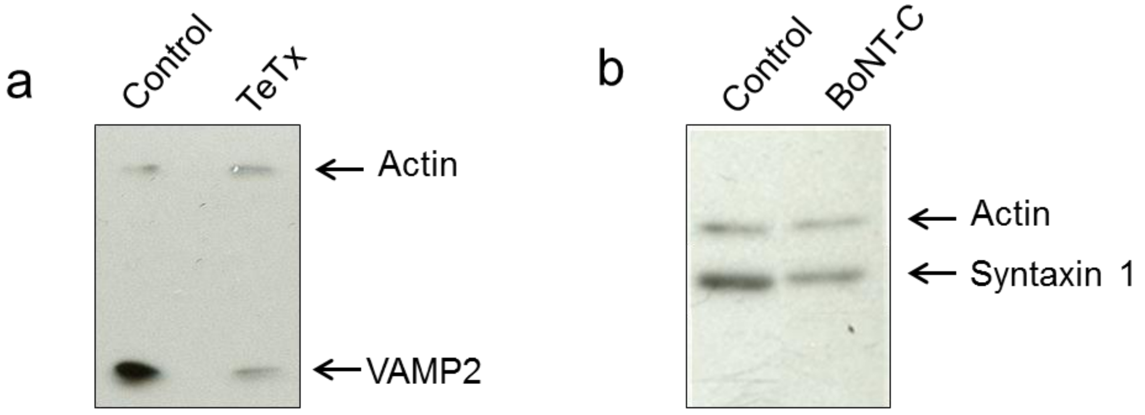

Figure S2) Expression of the light chains of TeTx and BoNT-C in MIN6 cells results in efficient cleavage of VAMP2 and Syntaxin 1. MIN6 cells were transiently transfected with the catalytic subunits of TeTx **(a)** or BoNT-C **(b)**. Two days later the cells were homogenized and the protein extracts analysed by western blotting using antibodies against Actin (loading control) and VAMP2 (a) or Syntaxin 1 (b). Taking in to account the transfection efficiency (about 50% in our experimental conditions), the toxins result in the effective cleavage of the SNARE proteins in the cells receiving the plasmids.

Text S1

Detailed explanation of the procedure used for the analysis of the different parameters of SLMVs recycling

Cells transfected with synaptophysin-pHluorin have been stimulated and imaged with TIRF and EPI illuminations (TIRFi and EPIi). The curves of the cocktail-evoked pHluorin fluorescence (pHF) signal represents at all times the net balance of exocytosis, endocytosis and re-acidification. If the cells are observed under EPIi (whole cell illumination) the increase of pHF reflects exocytosis (EXO(t)) and the decay of pHF, fusion pore closure and re-acidification (ENDO'(t)); under TIRFi instead, the fluorescence decay is determined by two different processes of the endocytic pathway: fusion pore closure followed by vesicle re-acidification (ENDO'(t)) and movement of the vesicle out of the evanescent wave field MOV(t). Thus, the pHF under EPIi represents EXO(t)-ENDO'(t) while under TIRFi it represents EXO(t)-ENDO'(t)-MOV(t). Bafilomycin A1 (BafA1), a v-type ATPase inhibitor, blocks the proton pump and prevents re-acidification, trapping synaptophysin-pHluorin-expressing vesicles in the fluorescent state. Thus, when BafA1 is present the re-acidification component is no longer contributing to the changes in the fluorescent signal. In this condition, the only component left under EPIi is EXO(t) and under TIRFi the fluorescence signal corresponds to EXO(t)-MOV(t). To obtain information about the kinetics of re-acidification and endocytosis we can compare EPI and TIRF curves that have been normalized to the maximum values measured with each EPI and TIRF methods, respectively. These values correspond to the maximal amount of vesicles which undergo fusion in each case. For EPIi, this value can be simply obtained by taking the maximum of the EPI trace after the stimulus in the presence of BafA1; in the case of TIRFi, the estimation of the maximum is more difficult because a fraction of the vesicles move out from the EW field because of the MOV(t). Thus, in this case to obtain the total number of fusion events, images collected in the first 30 s after the stimulus were superposed with Photoshop. Once determined the EPI and TIRF maximum values, we normalized the curves and proceeded with the analysis.
